# Supplementary material for: Ultrasound for the Detection of Inflammatory Abdominal Aortic Aneurysms: A Case and Validation Series
Source: Diagnostics (Basel). 2023 May 9;13(10):1669. doi: 10.3390/diagnostics13101669 (PMC10216955; doi:10.3390/diagnostics13101669)
Supplement: Supplementary file 1 [file diagnostics-13-01669-s001.zip › diagnostics-2349896-supplementary.pdf]

Ultrasound for the detection of inflammatory abdominal aortic aneurysms: a case and validation series.

Supplementary

Case series: individual ultrasound images

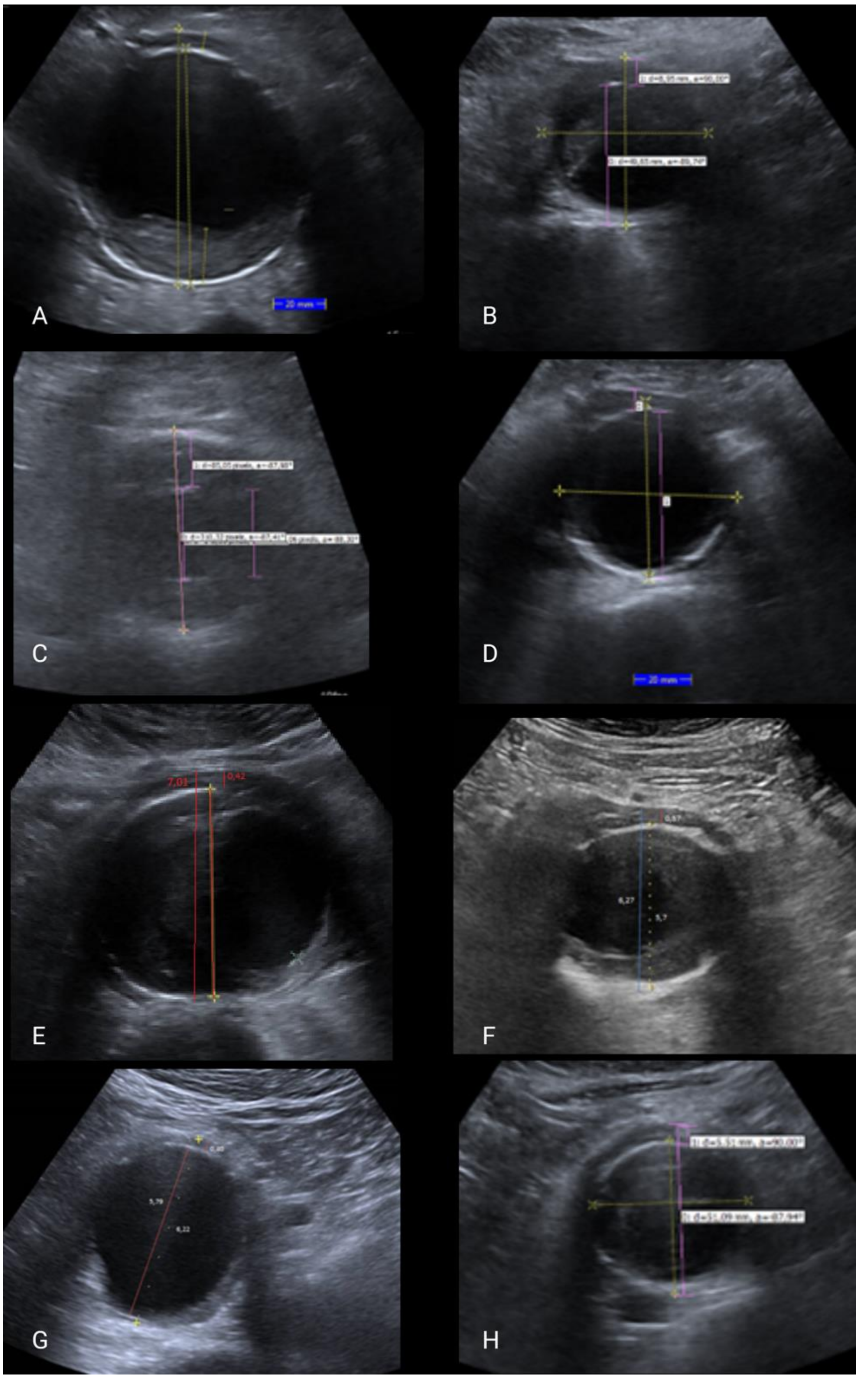

Figure S1a: positive ultrasound imaging in the case series group.

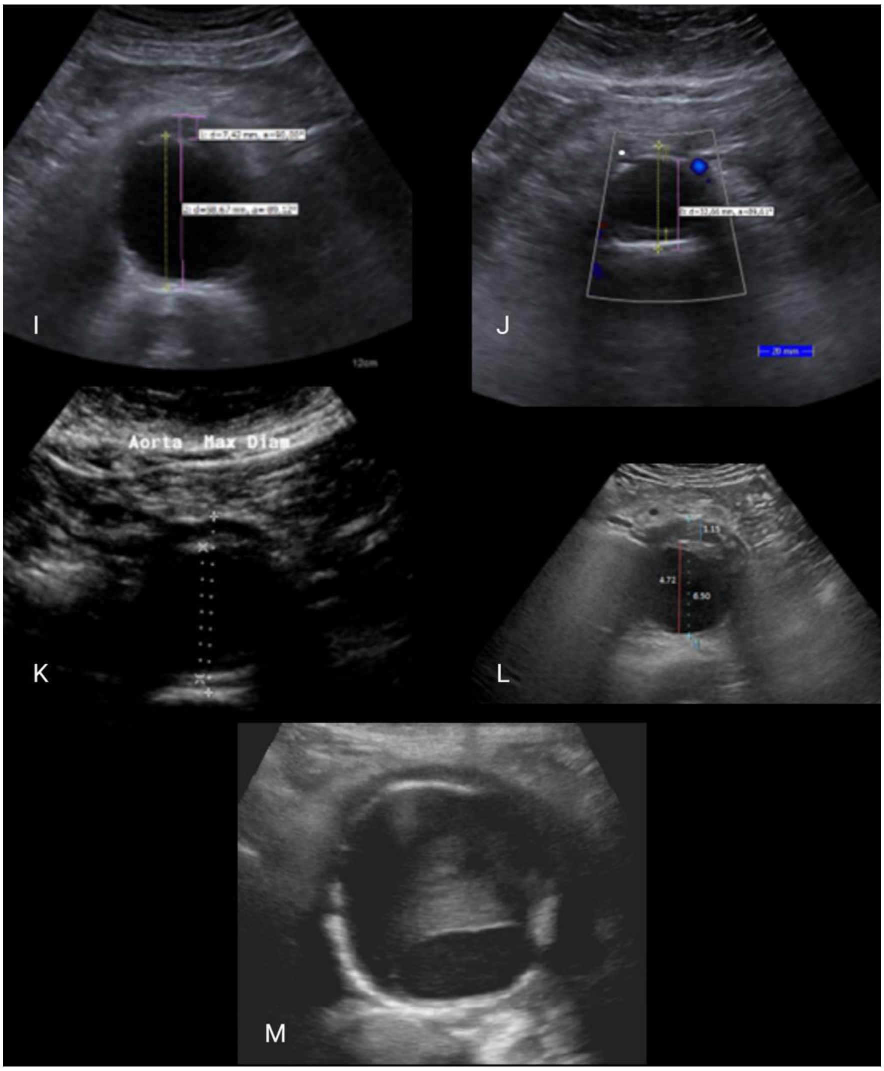

Figure S1b: positive ultrasound imaging in the case series group.

Feasibility series: individual ultrasound images

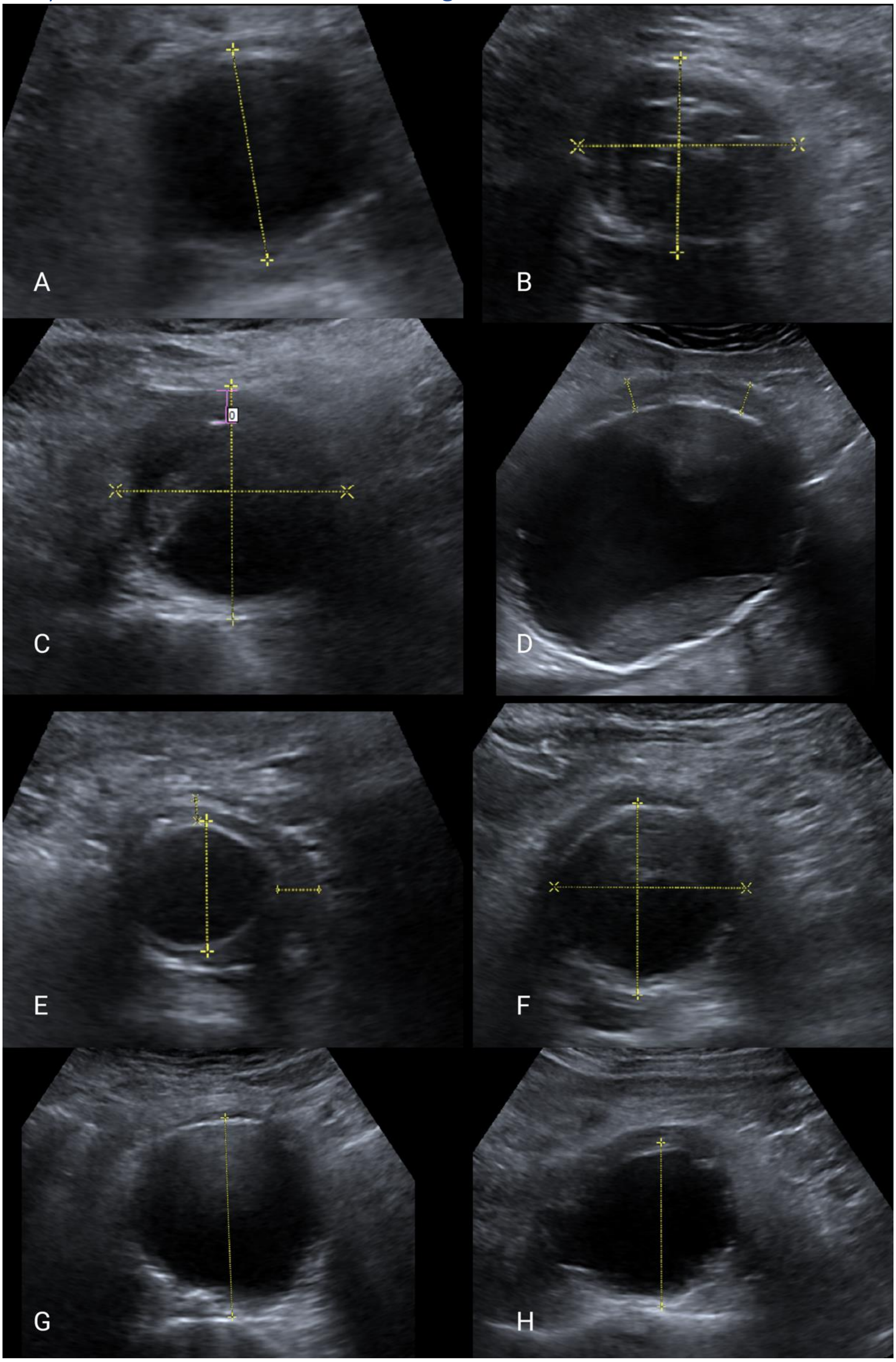

Figure S2: positive ultrasound imaging cases in the AAA group.
